# Decoding the structural and functional diversity of GABA_A_ receptors: from ensemble logic to therapeutic opportunities

**DOI:** 10.3389/fphar.2025.1697905

**Published:** 2025-10-15

**Authors:** Mario Treviño, Magdalena Guerra-Crespo, Francisco J. Padilla-Godínez, Luis O. Soto-Rojas, Elías Manjarrez, Emmanuel Ortega-Robles, Julieta Rodríguez-de Ita, Oscar Arias-Carrión

**Affiliations:** ^1^ Laboratorio de Plasticidad Cortical y Aprendizaje Perceptual, Instituto de Neurociencias, Universidad de Guadalajara, Guadalajara, Mexico; ^2^ Laboratory of Regenerative Medicine, Department of Physiology, Faculty of Medicine, National Autonomous University of Mexico, Mexico City, Mexico; ^3^ Department of Mathematics and Physics, Western Institute of Technology and Higher Education, Tlaquepaque, Mexico; ^4^ Laboratorio de Patogénesis Molecular, Facultad de Estudios Superiores Iztacala, Universidad Nacional Autónoma de México, Tlalnepantla, Mexico; ^5^ Instituto de Fisiología, Benemérita Universidad Autónoma de Puebla, Puebla, Mexico; ^6^ División de Neurociencias | Clínica, Instituto Nacional de Rehabilitación Luis Guillermo Ibarra Ibarra, Mexico City, Mexico; ^7^ Tecnologico de Monterrey, Escuela de Medicina y Ciencias de la Salud, Monterrey, Mexico; ^8^ Tecnologico de Monterrey, Escuela de Medicina y Ciencias de la Salud, Mexico City, Mexico

**Keywords:** GABA_A_ receptors, synaptic transmission, cryo–electron microscopy, inhibitory circuits, receptor assembly, neural plasticity

## Abstract

GABA_A_ receptors (GABA_A_Rs) are no longer viewed as uniform inhibitory switches but as structurally diverse, dynamically regulated ensembles that decode inhibitory signals with remarkable spatial and temporal precision. Their heterogeneity arises not only from the nineteen subunit genes but also from the combinatorial logic of assembly, alternative splicing, stoichiometry, post-translational modifications, and adaptive trafficking. These ensembles function as computational modules, tuned to the demands of individual circuits where they regulate excitability, gain control, and plasticity. Here, we highlight how recent advances in cryo–electron microscopy have transformed the field, revealing unexpected conformational states, novel ligand-binding pockets, and regulatory interfaces with accessory proteins, such as NACHO. *In vivo* studies demonstrate that individual neurons often co-express multiple receptor subtypes, forming heterogeneous ensembles that integrate inputs from GABA, neurosteroids, histamine, endocannabinoids, and exogenous ligands. This ensemble logic reframes inhibition as a circuit-specific computation rather than a uniform force. In this review, we discuss how disorders once attributed to “too little inhibition”—including epilepsy, chronic pain, schizophrenia, and Parkinson’s disease—can now be traced to disruptions in receptor assembly, trafficking, or ensemble composition. We also examine how classical pharmacology, with benzodiazepines and barbiturates as blunt instruments, falls short of capturing this complexity. By contrast, emerging approaches—subtype-selective allosteric modulators, gene editing, chaperone manipulation, and AI-guided ligand design—point toward precision therapeutics that recalibrate inhibition at the level of specific cell types, ensembles, and circuit motifs. Taken together, inhibition emerges not as a static force but as a flexible, ensemble-driven computation embedded in receptor structure and circuit architecture, and modulated by internal states and environmental context. Decoding this logic and learning to manipulate it with precision marks the next frontier in inhibitory neuroscience and the development of next-generation therapies for brain disorders.

## 1 Introduction

GABAergic inhibition remains one of the most powerful levers for sculpting brain activity, both in physiology and in therapy. Clinically, it underpins the management of anxiety, epilepsy, and insomnia, and provides the foundation of general anesthesia. At the molecular heart of these interventions lies the γ-aminobutyric acid type A receptor (GABA_A_Rs), a ligand-gated chloride channel responsible for the vast majority of fast inhibitory transmission in the mammalian brain. Benzodiazepines, barbiturates, and neurosteroids achieve their therapeutic effects through GABA_A_Rs. Nevertheless, most of these compounds were conceived in an era that predated our modern understanding of receptor diversity. Their success is shadowed by limitations—tolerance, paradoxical excitation, cognitive blunting, and addiction—that reflect the crude targeting of a receptor family far more diverse than once imagined (Bravo-Hernández et al., 2016; Sente, 2024).

GABA_A_Rs are not monolithic. They are heteropentameric assemblies, constructed from a family of nineteen homologous subunits (α1–6, β1–3, γ1–3, δ, ε, θ, π, and ρ1–3). The combinatorial rules that govern their assembly permit immense diversity, yet evolutionary and physiological constraints narrow the repertoire: only about 26 native assemblies have been experimentally confirmed, each conserved across vertebrate species, underscoring their biological significance (Burt and Kamatchi, 1991; Benke et al., 1996; Olsen and Sieghart, 2008; 2009; Sieghart and Savić, 2018; Scholze et al., 2020; Sadamitsu et al., 2021).

Still, subunit identity is only the opening chapter of this story. Advances in cryo–electron microscopy (cryo-EM) have provided near-atomic resolution of receptor architecture, revealing variations in stoichiometry, spatial configuration, and ligand-binding pockets that shatter the illusion of uniformity (Nakane et al., 2020; Sente, 2024). These discoveries elevate GABA_A_Rs from simple inhibitory switches to modular and plastic devices. Neurons, it turns out, do not commit to a single inhibitory receptor species; they co-express multiple subtypes, assembling them into dynamic ensembles that are reshaped by activity, hormones, injury, and inflammation (Franco-Enzástiga et al., 2022; Rodríguez-Palma et al., 2023). Endogenous modulators and accessory proteins weave yet another layer of complexity, allowing neurons to decode a chemically crowded extracellular milieu with exquisite temporal and spatial precision.

The consequences of this molecular heterogeneity reverberate across circuits. α5- and α6-containing receptors sustain tonic inhibition in the hippocampus and cerebellum, respectively, while also contributing to nociceptive regulation in spinal networks (Bravo-Hernández et al., 2016; Rodríguez-Palma et al., 2023). In basal ganglia loops, impaired assembly and altered chloride gradients drive motor dysfunction in Parkinson’s disease (Borgkvist et al., 2015; Lozovaya et al., 2018). In thalamocortical pathways, spinal GABA_A_Rs gate sensory throughput, linking the molecular diversity of inhibition to perception itself (Mahrous et al., 2025).

Particularly disruptive are the extrasynaptic receptors, enriched in α5, α6, or δ subunits. Their high GABA affinity and resistance to desensitisation position them as guardians of tonic inhibition. They are also uniquely vulnerable—and uniquely targetable. Epigenetic and genetic manipulations underscore their therapeutic potential: HDAC4 silencing recalibrates subunit expression and reduces GABA reuptake, thereby restoring inhibitory balance and suppressing seizures in animal models (Zhang Y. et al., 2019; Ianniello et al., 2025). In a distinct yet convergent pathway, the inhibition of microRNA-155 reinstates GABRA1 expression in glioblastoma (D’Urso et al., 2012) and normalises GABAergic tone in post-ischemic seizures by attenuating neuroinflammation and transporter overexpression (Zhang W. et al., 2019). Consistent with their specialised function, extrasynaptic receptors also display unique pharmacological fingerprints, including heightened sensitivity to Zn^2+^ and neurosteroids (Kasaragod et al., 2022).

Thus, GABAARs emerge as flexible, adaptive molecular computers. Their structural diversity, assembly logic, and circuit embedding conspire to generate inhibition that is both context-dependent and state-dependent, as well as plastic. They do not merely silence—they compute. They set thresholds for memory, refine motor precision, gate sensory flow, and calibrate pain and mood.

In this review, we propose a disruptive reframing: GABA_A_Rs must be understood not as uniform inhibitory gates but as dynamic ensembles, embedded in circuits and continuously recalibrated by molecular and environmental cues. By weaving structural biology, receptor assembly, ensemble logic, and systems neuroscience into a single framework, we argue that inhibition itself is a computation—one that can be targeted, biased, and potentially reprogrammed. This shift opens a therapeutic horizon in which inhibition is no longer globally suppressed, but precisely tuned at the molecular and circuit levels.

## 2 GABA_A_ receptor architecture and function

**FIGURE 1:**
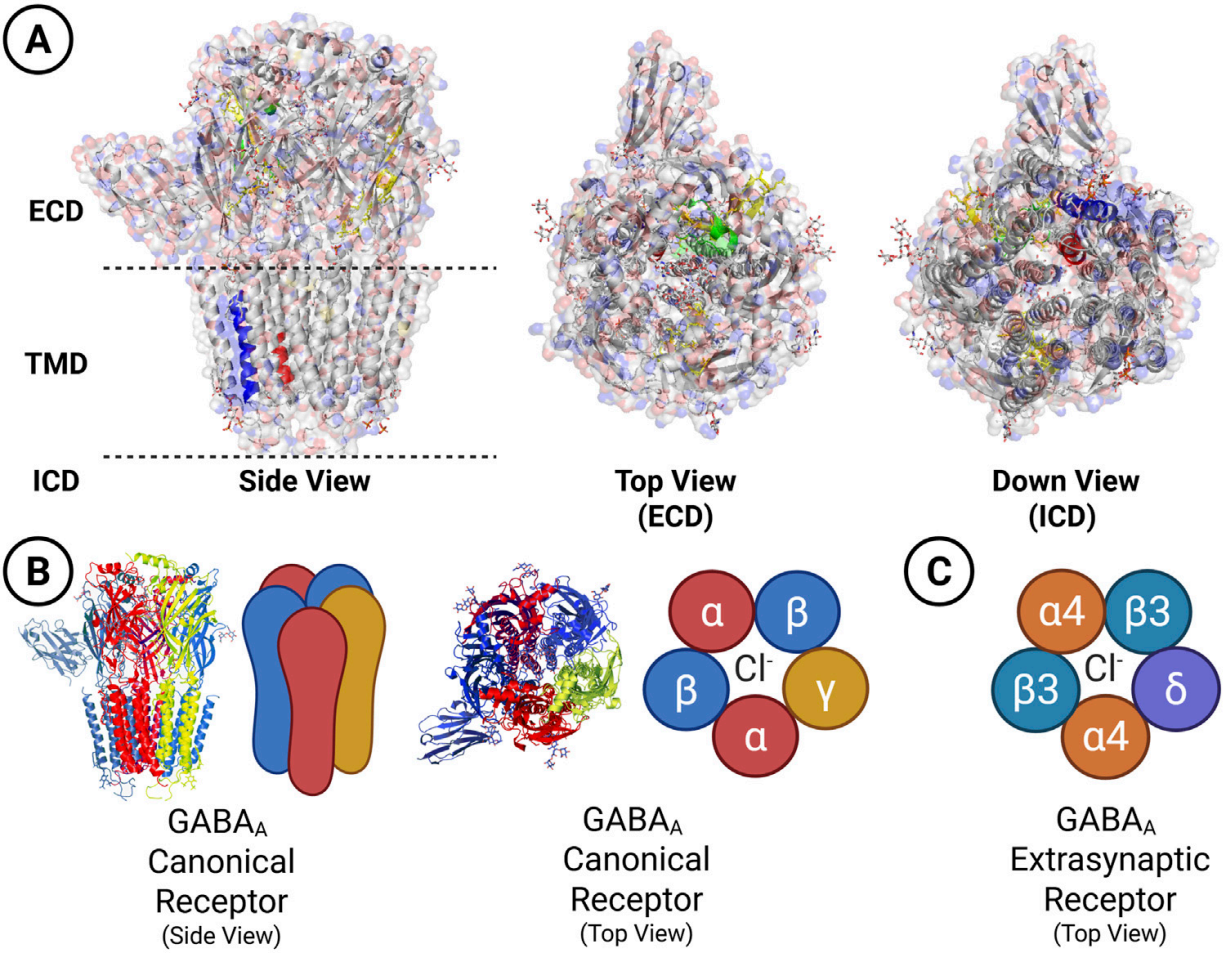
Structural organisation and binding sites of canonical and noncanonical GABA_A_ receptors. **(A)** Ribbon representation of the canonical α1β3γ2 receptor (2α1, 2β3, 1γ2) generated from cryo-EM coordinates (PDB ID: 6I53; Laverty et al., 2019). Side, top, and bottom views are shown. Structural domains are highlighted: extracellular domain (green), transmembrane domain (blue), and chloride pore (red). Receptor structures were visualised and rendered using molecular graphics software (PyMOL). **(B)** Subunit arrangement and binding sites of canonical and noncanonical receptors. Canonical α1β3γ2 subunits are colour-coded (α1 in red, β3 in blue, γ2 in yellow). GABA-binding sites are located at β–α interfaces, and the benzodiazepine site at the α–γ interface (Kasaragod et al., 2022). **(C)** Noncanonical α4β3δ configuration representative of extrasynaptic receptors mediating tonic inhibition, with subunits individually colour-coded. A neurosteroid-binding site in the transmembrane domain is indicated (Carver and Reddy, 2013; Masiulis et al., 2019). Endogenous modulators, such as taurine and netrin, as well as accessory complexes like the TMEM132B–GABA_A_ receptor, further diversify extrasynaptic receptor function within ensemble signalling. Structural schematics were created with BioRender.com.

GABA_A_Rs are the primary mediators of fast inhibitory neurotransmission in the mammalian brain. They assemble as heteropentameric chloride channels, built from a gene family encoding nineteen variants: α1–6, β1–3, γ1–3, δ, ε, θ, π, and ρ1–3 (Barnard et al., 1998; Sieghart et al., 2022). Although theoretical combinations reach into the hundreds of thousands, evolutionary and physiological constraints—including subunit compatibility, developmental timing, and subcellular localisation—restrict the repertoire observed *in vivo*. Approximately 26 native assemblies have been reported, classified as “identified,” “probable,” or “tentative” depending on experimental support (Burt and Kamatchi, 1991; Benke et al., 1996; Olsen and Sieghart, 2008; 2009; Sieghart and Savić, 2018; Scholze et al., 2020). The most common configuration consists of two α subunits, two β subunits, and one γ subunit, a stoichiometry confirmed across multiple studies (McKernan et al., 1991; Benke et al., 1996; Tretter et al., 1997) (Figure 1).

The subunit composition determines the receptor’s pharmacological profile, cellular localisation, and the mode of inhibition it mediates. Synaptic and extrasynaptic receptors differ profoundly in structure–function properties, giving rise to distinct inhibitory regimes (Bravo-Hernández et al., 2016; Rodríguez-Palma et al., 2023). Synaptic GABA_A_Rs, typically assembled from α1–3 and γ2 subunits, mediate phasic inhibition. These receptors have low GABA affinity, desensitise rapidly, and generate brief yet powerful inhibitory postsynaptic currents, with unitary conductances exceeding 20 pS in hippocampal neurons (Yeung et al., 2003; Lee and Maguire, 2014). In contrast, extrasynaptic GABA_A_Rs, enriched in α4–6 and δ subunits, sustain tonic inhibition. They exhibit high GABA affinity, minimal desensitisation, and persistent low-amplitude currents, with unitary conductances of ∼12 pS in hippocampal neurons and around 14 pS in cerebellar granule cells (Leao et al., 2000; Yeung et al., 2003; Lee and Maguire, 2014; Kasaragod et al., 2022).

At the molecular level, GABA binds to orthosteric sites at β–α subunit interfaces, triggering conformational changes that open the central chloride pore. The resulting influx hyperpolarises the postsynaptic membrane and suppresses neuronal excitability (Pressey et al., 2023). Subunit identity shapes not only GABA affinity and desensitisation kinetics, but also the receptor’s responsiveness to pharmacological modulators, including benzodiazepines, neurosteroids, barbiturates, and anaesthetics (Sigel et al., 1990; Verdoorn et al., 1990; Ghit et al., 2021). These pharmacological distinctions are summarised in Table 1.

**TABLE 1:** Pharmacological agents acting on GABA_A_ receptors.

Agent/Class	Binding site/Mechanism	Subunit selectivity	Clinical/Functional applications	References
GABA	Orthosteric agonist at β–α interface	All subtypes	Initiates chloride influx; baseline inhibitory signalling	Pressey et al. (2023)
Benzodiazepines	Positive allosteric modulators (PAMs) at the canonical α–γ2 interface; also interact with noncanonical α–β and β–γ interfaces, and transmembrane sites	High-affinity: α1–3, α5 + γ2 (γ2 essential); non-canonical sites also in β2- and δ-containing receptors	Sedation, anxiolysis, anticonvulsion, muscle relaxation; subtype-selective ligands explored for cognition (α5 NAMs, e.g., basmisanil), schizophrenia, depression, pain	Carver and Reddy, 2013; Masiulis et al., 2019; Rudolph and Möhler, 2014; Sigel and Ernst, 2018
Barbiturates	PAM at the transmembrane domain; direct agonism at high doses	α, β, γ (β2/3 preference)	Sedation, anaesthesia, and epilepsy prolong channel open time	Carver and Reddy (2013)
Neurosteroids (e.g., allopregnanolone)	PAM/NAM at transmembrane sites	δ > γ2-containing	Depression, anxiety, epilepsy, and postpartum disorders enhance tonic inhibition	Legesse et al., 2023; Maguire and Mody, 2007
THIP (gaboxadol)	Orthosteric agonist	δ-containing (α4β3δ, α6β3δ)	Enhances tonic inhibition; experimental for epilepsy, sleep disorders	Wongsamitkul et al., 2016; Falk-Petersen et al., 2017
DS2	δ-selective PAM	α4β3δ, α6β3δ	Enhances tonic inhibition; experimental for epilepsy, anxiety	Falk-Petersen et al. (2017)
S44819	α5-selective NAM	α5-containing	Antidepressant effects: reduces cognitive impairment	Etherington et al. (2017)
GL-II-73	PAM at benzodiazepine site (α5-preferring)	α5-containing	Antidepressant, anxiolytic, pro-cognitive effects	Prevot et al., 2019; Guet-McCreight et al., 2024
Bicuculline	Competitive antagonist at the GABA site	All subtypes	Blocks phasic inhibition; experimental tool	Sigel et al. (1990)
Gabazine (SR-95531)	Competitive antagonist at the GABA site	All subtypes	Isolates tonic inhibition; experimental tool	Sigel et al. (1990)
Picrotoxin	Noncompetitive channel blocker	All subtypes	Convulsant; blocks chloride conductance	Verdoorn et al. (1990)
Zn^2+^	Noncompetitive allosteric blocker	αβ (extrasynaptic) > γ2	Inhibits tonic inhibition; modulates pain, seizures	Kasaragod et al. (2022)
L-655,708	α5-selective inverse agonist	α5-containing	Enhances cognition; experimental for memory disorders	Prevot et al. (2019)
Pregnenolone sulfate	NAM; pore blocker	ρ1-containing	Modulates sensory processing, thalamocortical inhibition	Carver and Reddy (2016)
β-Estradiol	NAM at the allosteric site	ρ-subunit receptors	Modulates visual, cognitive circuits	Chuang and Reddy (2018)
Loreclezole	PAM at β2/3 sites	β2, β3	Experimental anticonvulsant	Olsen and Sieghart (2008)
Furosemide	Inhibitor of tonic inhibition	α4/α6-containing	Sensory modulation: experimental tool	Loeza-Alcocer et al., 2013; Kasaragod et al., 2022
Endocannabinoids	PAMs at the β2 M4 site that enhance low-GABA currents; 2-AG and NA-glycine act at overlapping sites	Require β2 subunit; active at δ-extrasynaptic receptors; NA-glycine is more potent than 2-AG but far less abundant	Modulate tonic inhibition; synergize with neurosteroids (THDOC) and diazepam; produce sedation/hypomotility independent of CB1/CB2 receptors	Sigel et al., 2011; Baur et al., 2013

Cryo–electron microscopy (cryo-EM) has resolved receptor architecture at near-atomic resolution, revealing the organisation of ligand-binding domains, allosteric sites, and the transmembrane pore (Laverty et al., 2019; Masiulis et al., 2019; Nakane et al., 2020; Sente, 2024). These studies illuminate structural features—hydrogen-bond networks, lipid interactions, and structured water molecules—that govern gating and drug binding (Phulera et al., 2018; Nakane et al., 2020). Subunit-specific pharmacology arises from this architecture. Benzodiazepines require a γ2 subunit and bind at the α–γ interface, whereas neurosteroids such as allopregnanolone act through transmembrane domains, showing enhanced efficacy at δ-containing receptors (Carver and Reddy, 2013; Masiulis et al., 2019; Legesse et al., 2023; Thompson, 2024). These preferences explain why neurosteroids strongly modulate tonic inhibition and stress responsiveness.

Innovative photoaffinity labelling has significantly enhanced our understanding of neurosteroid binding. High-affinity steroid pockets at β+–α− interfaces, essential for modulating 3α-hydroxylated neurosteroids, were first identified by Jayakar and colleagues (Jayakar et al., 2020). Subsequent work confirmed this site’s specificity using photoreactive analogues and competitive inhibition assays (Wu et al., 2019). Earlier photoprobes refined site mapping and distinguished steroid-binding domains from those of barbiturates or etomidate (Savechenkov et al., 2017). Notably, work with photoreactive allopregnanolone analogues has revealed overlapping and distinct steroid-binding sites in nicotinic receptors, underscoring the conserved design principles across pentameric ligand-gated ion channels (Yu et al., 2019). Collectively, these studies provide a molecular framework for understanding the divergent actions of neurosteroids on GABA_A_Rs.

Post-translational modifications add another layer of control, regulating receptor trafficking and surface stability. Phosphorylation, palmitoylation, and ubiquitination influence synaptic clustering and turnover, thereby modulating inhibitory tone (Lorenz-Guertin and Jacob, 2018). Pathological states often exploit these pathways: mutations in α1 or γ2 subunits accelerate endocytosis, reducing inhibitory efficacy in epilepsy (Bradley et al., 2008). Chaperones and accessory proteins orchestrate the folding and insertion of subunits. For example, α1-containing receptors exhibit distinct trafficking compared to α4/δ receptors, which preferentially localise extrasynaptically and display enhanced sensitivity to neurosteroids (Ghit et al., 2021). These mechanisms are elaborated further in Section 4 on receptor assembly.

A broad toolkit supports functional characterisation. Heterologous systems, such as HEK293 cells and *Xenopus* oocytes, enable the systematic testing of subunit combinations (Sigel et al., 1990; Verdoorn et al., 1990; Baur et al., 2006; Kaur et al., 2009; Sadamitsu et al., 2021). Subunit concatenation and structural modelling provide precise control over stoichiometry (Sente, 2024), while single-cell RNA sequencing reveals native expression patterns. Unnatural amino acid mutagenesis expands the toolkit, allowing chemical substitutions to probe binding and activation (Padgett et al., 2007; Hanek et al., 2010; Lummis et al., 2011). These approaches are summarised in Table 2.

**TABLE 2:** Experimental approaches to study GABA_A_ receptor subtypes.

Method	Target/Approach	Subunit specificity	Application/Outcome	References
Subtype-selective agonists	THIP (gaboxadol), DS2	δ-containing (α4β3δ, α6β3δ)	Enhances tonic inhibition; screens for epilepsy, anxiety drugs	Wongsamitkul et al., 2016; Falk-Petersen et al., 2017
Canonical and noncanonical amino acid incorporation	Site-specific substitutions in binding interfaces	Any subunit	Probes ligand binding residues, gating transitions	Padgett et al., 2007; Hanek et al., 2010; Lummis et al., 2011
Subunit concatenation	Genetically linked subunits	Defined stoichiometries	Controls assembly order; resolves pharmacology of isoforms	Sente, 2024; Baur et al., 2006
Photoreactive molecules	Ligand/pore-binding analogues with photolabels	Subunit-dependent	Maps binding sites and conformational dynamics	Qiao et al., 2022; Maleeva et al., 2024; Lopez et al., 2025
Competitive antagonists	Bicuculline, gabazine	All subtypes	Distinguishes phasic vs. tonic inhibition; maps synaptic currents	Sigel et al. (1990)
Allosteric inhibitors	Zn^2+^, α-cobratoxin	αβ, α1/α3	Probes extrasynaptic function; modulates pain, seizures	Kasaragod et al., 2022; Kudryavtsev et al., 2015
Site-directed mutagenesis	Point mutations in ligand/transmembrane sites	Any subunit	Identifies key residues for ligand binding and gating	Sieghart et al., 1999; Luger et al., 2015; Sugasawa et al., 2020
Recombinant expression systems	HEK293 cells, *Xenopus* oocytes	Any combination	Reconstitutes assemblies; assesses kinetics	Sigel et al., 1990; Hartiadi et al., 2016
Electrophysiology	Patch-clamp, single/whole-cell recordings	All subtypes	Quantifies kinetics, desensitisation, and drug effects	Verdoorn et al. (1990)
Subtype-selective blockers	L-655,708 (α5-inverse agonist)	α5-specific	Enhances hippocampal cognition; memory-enhancing drug testing	Prevot et al., 2019; Guet-McCreight et al., 2024
Structural studies	Cryo-EM, X-ray crystallography	All assemblies	Resolves ligand-binding sites and conformational states	Masiulis et al., 2019; Laverty et al., 2019
Optogenetic pharmacology	LiGABARs, photoswitchable agonists	Subtype-specific (α5, γ2)	Enables millisecond control of inhibition; targets epilepsy	Lin et al. (2015)
Single-cell RNA sequencing	Transcriptomic profiling	All subunits	Maps subunit expression across brain regions and cell types	Sequeira et al. (2019)

These molecular insights are already shaping the field of drug discovery. Subtype-selective ligands targeting α2- or α3-containing receptors demonstrate anxiolytic efficacy without the sedative profile of classical benzodiazepines (Rudolph and Knoflach, 2011). More recently, photopharmacological strategies have enabled the reversible, light-controlled modulation of receptor subtypes *in vivo* (Qiao et al., 2022; Maleeva et al., 2024; Lopez et al., 2025). Translating receptor-specific modulation into circuit-level precision remains challenging, yet it holds promise for next-generation therapies.

Ultimately, the diversity of GABA_A_Rs reflects not only gene expression but also a multilayered process of dynamic assembly, trafficking, and integration into specific domains. Understanding how subunit composition and post-translational regulation interact to sculpt inhibitory tone is essential for bridging molecular structure with circuit function. As high-resolution datasets accumulate, the central challenge will be to synthesise these insights into circuit-aware models of inhibition—frameworks that guide therapeutic efforts aimed at restoring inhibitory precision in disease states.

## 3 Structural diversity: more than subunit identity

The classical framework for understanding GABA_A_Rs diversity has long rested on the principle of subunit identity as the central determinant of receptor function. Each of the nineteen subunits—α1–6, β1–3, γ1–3, δ, ε, θ, π, and ρ1–3—was thought to imprint distinct pharmacological and biophysical features upon assembled receptors, thereby dictating their localisation, ligand sensitivity, and inhibitory profile (Barnard et al., 1998; Farrant and Nusser, 2005; Carver and Reddy, 2013; Masiulis et al., 2019; Kasaragod et al., 2022; Sieghart et al., 2022; Thompson, 2024). Within this model, synaptic receptors composed of α and γ subunits mediated phasic inhibition, while extrasynaptic receptors enriched in α and δ subunits conferred tonic inhibition. This deterministic, subunit-centric view provided an elegant organising principle; however, mounting evidence reveals it to be incomplete.

Recent structural and functional advances—particularly those achieved through cryo–electron microscopy—demonstrate that even receptors built from identical subunits can diverge in stoichiometry and spatial arrangement, yielding isoforms with distinct signalling properties (Sente, 2024). These discoveries dismantle the assumption that subunit composition alone dictates receptor identity. Instead, they portray GABA_A_Rs as modular, plastic molecular assemblies whose architecture can be reconfigured, allowing neurons to fine-tune inhibition across contexts and timescales.

At the heart of receptor function lies the canonical binding and gating process. Classical GABA_A_Rs contain two orthosteric GABA-binding sites at β–α interfaces. Occupation of a single site is sufficient to induce partial channel activation, but simultaneous occupancy of both sites drives the conformational transitions that maximise opening, prolong burst duration, and enhance chloride conductance, resulting in robust phasic inhibition (Amin and Weiss, 1993; Mortensen et al., 2012). When a γ subunit is present, the receptor gains a third site at the α–γ interface, which serves as the allosteric locus for benzodiazepine action. Here, ligands act not as agonists but as positive allosteric modulators, enhancing receptor function without directly opening the channel. Beyond these canonical architectures, unconventional receptor assemblies have been described. Some hybrid configurations incorporate noncanonical interfaces responsive to histamine, suggesting that certain receptor forms integrate signals from multiple neurotransmitter systems (Sente, 2024). Benzodiazepines, once thought to act exclusively at the α–γ2 interface, also engage additional noncanonical sites at α–β and β–γ interfaces, broadening the pharmacological landscape (Sigel and Ernst, 2018). Together, these findings support a provocative reconceptualisation: GABA_A_Rs can act as coincidence detectors, integrating multiple chemical signals to generate context-specific inhibitory outputs.

Computational modelling magnifies this picture of diversity. Early estimates placed the number of possible GABA_A_Rs configurations near 800 (Barnard et al., 1998). Contemporary modelling now suggests over 324,000 potential receptor assemblies, each with distinct conductance, desensitisation kinetics, and ligand sensitivity (Sente, 2024). These simulations demonstrate that subtle differences in subunit composition can significantly alter channel behaviour. For example, inclusion of a γ2 subunit increases conductance, while β2 subunits accelerate desensitisation (Sigel et al., 1990; Verdoorn et al., 1990; Ghit et al., 2021). Structural diversity also sculpts receptor pharmacology, trafficking, and localisation. Receptors composed of α4/δ subunits respond to neurosteroids in ways that differ fundamentally from α1/γ2 receptors, and they preferentially localise to extrasynaptic membranes, where they sustain tonic inhibition (Sieghart et al., 1999; Sieghart and Savić, 2018; Ghit et al., 2021). These examples illustrate how molecular diversity reverberates at the circuit level.

From a systems perspective, this architectural plasticity supports the emergence of signaling ensembles—heterogeneous collections of GABA_A_Rs with distinct pharmacological and spatial properties. These ensembles enable neurons to decode complex mixtures of ligands, such as GABA, histamine, and neurosteroids, and to generate inhibitory responses that are tuned to region, state, and demand (Rodríguez-Palma et al., 2023; Sente, 2024). Endogenous modulators add further richness: taurine enhances inhibitory control (Jia et al., 2008), netrin influences receptor function during development and plasticity (Chan et al., 2022), and accessory proteins such as TMEM132B form complexes with GABA_A_Rs to diversify ensemble behaviour, with implications for circuit plasticity and behaviors such as alcohol consumption (Wang G. et al., 2024). In this light, inhibition ceases to be a uniform process and becomes instead a computation, implemented through the “ensemble logic” of receptors that dynamically adjust inhibitory tone across circuits, behavioural states, and disease conditions.

Despite this progress, vast areas remain unexplored. Many predicted receptor isoforms and stoichiometries have not been experimentally confirmed, and the physiological consequences of multi-ligand binding remain elusive. One obstacle lies in the pharmacological toolkit, which lacks the resolution to distinguish subtle structural variants. Closing this gap requires integrative approaches, including high-resolution single-particle cryo-EM to capture native structures *in situ*, proteomic methods to catalogue receptor assemblies, and structural modelling to map their conformational landscapes. Molecular dynamics simulations have already advanced this frontier, revealing ligand-binding pathways, gating transitions, and allosteric mechanisms at atomistic resolution (Zou et al., 2019; Pieroni et al., 2023; Yu et al., 2024; Haloi et al., 2025). Approaches such as adaptive sampling, Markov state modelling, and high-performance computing complement experimental work, not only validating observed structures but also guiding rational drug design. By pinpointing allosteric sites and predicting biased signalling outcomes, computational models now illuminate pathways to therapies that selectively modulate inhibition with unprecedented precision.

## 4 Assembly mechanisms: the role of NACHO and beyond

**FIGURE 2:**
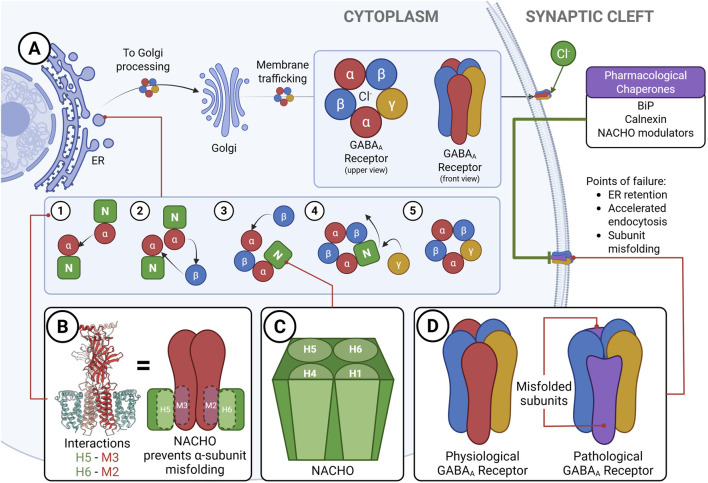
Assembly pathway and quality control of GABA_A_ receptors. **(A)** Schematic of GABA_A_ receptor biogenesis in the endoplasmic reticulum (ER), highlighting the stepwise incorporation of subunits stabilised by the chaperone NACHO (N). Assembly intermediates proceed through ER quality control, Golgi processing, and membrane trafficking to form functional chloride channels at the plasma membrane. Failure points include ER retention, accelerated endocytosis, or subunit misfolding, processes modulated by accessory chaperones such as BiP, calnexin, and pharmacological NACHO modulators. **(B)** Structural depiction of NACHO interactions with α-subunit transmembrane helices (H5–M3 and H6–M2), preventing misfolding during early receptor assembly (Hooda et al., 2024, preprint). **(C)** Schematic representation of the NACHO scaffold, stabilising α subunits and guiding sequential incorporation of β and γ subunits. **(D)** Comparison of physiological and pathological receptors, showing the consequences of subunit misfolding for receptor architecture. Together, these mechanisms illustrate how NACHO and its associated chaperones regulate receptor biogenesis, ensuring the fidelity of GABAergic inhibition and preventing the pathological disruption of inhibitory tone. Schematics were created with BioRender.com.

The assembly of GABA_A_Rs is a tightly regulated, multistep process that ensures the precise selection, folding, and integration of subunits into functional pentameric channels. With nineteen subunit isoforms available, neurons face the formidable challenge of coordinating compatibility rules, subunit stoichiometry, and trafficking pathways to preserve inhibitory homeostasis when this process is disrupted—whether by genetic mutations, cellular stress, or regulatory failure—the resulting defects in receptor function compromise inhibitory tone and contribute to the development of pathological states (Figure 2).

### 4.1 Chaperones and the stepwise assembly process

Recent cryo–electron microscopy studies have shed light on the earliest phases of receptor biogenesis. Established work has long indicated that distinct GABA_A_Rs subtypes, including α5- and α6-containing receptors, contribute to tonic inhibition and often localise extrasynaptically. Their biogenesis appears to involve specialised assembly routes and accessory proteins that safeguard correct folding, trafficking, and targeting (Bravo-Hernández et al., 2016; Sieghart et al., 2022).

Structural models of assembly intermediates, informed by recent work on the nicotinic acetylcholine receptor chaperone (NACHO), now suggest how receptor configurations emerge. Evidence indicates that NACHO may bind to α subunits, stabilising early intermediates by shielding hydrophobic interfaces from misfolding and guiding the sequential incorporation of β subunits, followed by γ or δ partners (Hooda et al., 2024). Because these data derive from a preprint, they remain provisional and require independent replication and peer-reviewed validation. In this model, NACHO functions as an intramembrane assembly factor, introducing regulatory checkpoints that ensure the ordered biogenesis of receptors. Additional cofactors, yet to be identified, are likely required for subunit selection, surface trafficking, and recycling dynamics. These processes appear sensitive to neuronal activity, hormonal state, and cellular stress, underscoring the idea that receptor composition is not static but dynamically responsive to physiological context (Saliba et al., 2007; Bradley et al., 2008; Merlaud et al., 2022; Yuan et al., 2024).

Although NACHO has emerged as a central assembly scaffold, it almost certainly does not act alone. Other cofactors, perhaps responsive to hormonal, metabolic, or epigenetic cues, are expected to influence folding efficiency, subunit availability, and exit from the endoplasmic reticulum (ER). Identifying these factors will be essential for understanding how neurons maintain the delicate balance of receptor subtypes in both physiological and pathological settings.

Failures in receptor assembly can impair inhibitory tone even in the absence of transcriptional changes. While altered subunit expression has been documented in epilepsy, Parkinson’s disease, autism spectrum disorders, and schizophrenia (Rudolph and Möhler, 2014; Lozovaya et al., 2018; Rodríguez-Palma et al., 2023), defects in folding, stoichiometry, or trafficking may escape detection by transcriptomic profiling. In Parkinson’s disease, for example, GABA_A_R subunit expression exhibits striking regional specificity, with reduced expression in thalamic nuclei and increased expression in cortical and basal ganglia circuits, thereby contributing to enhanced GABAergic tone and impaired motor control (Alharbi et al., 2024). Whether such changes reflect transcriptional plasticity or breakdowns in receptor biogenesis remains unresolved, but they carry profound therapeutic implications. Targeting the molecular checkpoints of receptor assembly may allow interventions not only at the level of mature receptor modulation but at the biosynthetic stage, biasing receptor output toward beneficial subtypes.

These insights have sparked growing interest in pharmacological chaperones—small molecules that stabilise folding intermediates and enhance the surface expression of receptors. Such “molecular glues” have already demonstrated therapeutic potential in other multimeric protein systems (Rui et al., 2023). Within the GABA_A_Rs field, strategies to modulate chaperones such as BiP (binding immunoglobulin protein), calnexin (a membrane-bound chaperone essential for glycoprotein folding), or NACHO itself represent an innovative frontier (Fukata and Fukata, 2010; Hooda et al., 2024, preprint; Wang Y.-J. et al., 2024). This approach signals a paradigm shift: from static modulation of mature receptors to dynamic regulation of the intracellular pathways that assemble them. Nevertheless, major challenges remain, including the development of real-time imaging tools to follow receptor assembly in live cells and the integration of these molecular events with circuit-level outcomes.

### 4.2 Membrane trafficking and dynamic receptor regulation

Assembly culminates in the formation of pentamers, but the life cycle of the receptor does not end there. GABA_A_Rs must be trafficked to the appropriate membrane domains and continuously regulated according to physiological demand. These processes include activity-dependent endocytosis, recycling, and reinsertion at synaptic sites (Saliba et al., 2007; Bradley et al., 2008; Merlaud et al., 2022). Receptor turnover is conformation-dependent, with active, desensitised, and resting states each exhibiting distinct internalisation and degradation rates. Pathological mutations exacerbate these dynamics: epilepsy-linked variants in α1 or γ2 subunits, for example, accelerate endocytosis and reduce synaptic inhibition (Bradley et al., 2008).

The advent of advanced tools has enabled direct visualisation of these processes. Dual-tagged γ2 subunits, such as γ2-pHFAP (a genetically engineered variant tagged with a fluorogen-activating peptide), allow real-time imaging of receptor trafficking in live neurons. These studies reveal that seizures accelerate receptor turnover and lysosomal degradation while simultaneously activating compensatory reinsertion mechanisms aimed at restoring inhibitory balance (Lombardi et al., 2020).

### 4.3 Post-translational regulation and accessory proteins

Post-translational modifications add further layers of regulation, refining the localisation, stability, and function of receptors. Phosphorylation, palmitoylation, SUMOylation, and ubiquitination govern clustering at synapses, membrane retention, and anchoring to scaffolding proteins (Lorenz-Guertin and Jacob, 2018). For example, phosphorylation modulates receptor binding to gephyrin, while palmitoylation by GODZ (Golgi-specific DHHC zinc finger protein) stabilises α5- and δ-containing receptors at extrasynaptic sites.

Accessory proteins exert equally critical roles. LH4 promotes clustering of γ2-containing receptors through interactions with neuroligin-2. Clptm1, an ER-resident protein, retains immature receptors within the ER, thereby restricting surface expression. Shisa7, a transmembrane protein, modulates benzodiazepine sensitivity and receptor decay kinetics; its deletion reduces benzodiazepine efficacy *in vivo* (Han et al., 2021). Together, these accessory factors act as a molecular interface between receptor assembly and circuit integration, enabling inhibitory tone to adapt dynamically to experience and physiological state.

### 4.4 Receptor assembly as a dynamic regulatory layer

Taken together, these findings reframe receptor biogenesis and trafficking as more than background processes. They emerge as an active regulatory layer in inhibitory signalling, dictating when, where, and how inhibition is expressed across neural circuits. By shaping subunit selection, folding, trafficking, and turnover, assembly mechanisms generate the diversity necessary for flexible inhibition in health and disease. This perspective opens therapeutic possibilities: targeting the assembly and trafficking machinery could recalibrate inhibitory tone with spatial and temporal precision unmatched by conventional pharmacology. Key accessory proteins and post-translational regulators that influence the assembly, trafficking, and function of GABA_A_Rs are summarised in Table 3.

**TABLE 3:** Accessory proteins and post-translational mechanisms regulating GABA_A_.

Regulatory factor	Type	Target subunits/Sites	Functional role	Implications for disease/Therapy	References
NACHO (TMEM35A)	Chaperone	α1, α5, α6	Stabilises assembly intermediates; promotes pentamer formation	Enhances extrasynaptic receptor expression; therapeutic target	Hooda et al., 2024*
BiP/Calnexin	ER-resident chaperones	Glycoprotein subunits	Ensures proper folding; retains misassembled receptors	Pharmacological chaperones for epilepsy	Fukata and Fukata (2010)
Gephyrin	Anchoring scaffold	γ2, α2–3 (synaptic)	Clusters of synaptic GABA_A_Rs support synapse formation	Mutations linked to epilepsy, autism	Lorenz-Guertin and Jacob (2018)
Shisa7	Transmembrane modulator	γ2-containing	Regulates benzodiazepine sensitivity, synaptic kinetics	Deletion reduces drug efficacy	Han et al. (2021)
Clptm1	ER retention protein	Immature subunits	Prevents premature trafficking	Dysregulation impairs tonic inhibition	Han et al. (2021)
LH4 (Lhfpl4)	Synaptic organiser	γ2-containing via neuroligin-2	Promotes synaptic clustering	Implicated in synaptic dysfunction	Han et al. (2021)
Palmitoylation (GODZ)	Lipid modification	α5, δ	Enhances extrasynaptic localisation	Modulates neurosteroid responsiveness	Lorenz-Guertin and Jacob (2018)
Phosphorylation	Reversible PTM	β3, γ2	Regulates endocytosis and synaptic anchoring	Altered in epilepsy, stress plasticity	Bradley et al. (2008)
Ubiquitination SUMOylation	Protein turnover/trafficking	Multiple subunits	Controls degradation, synaptic retention	Regulates receptor abundance in stress and inflammation	Merlaud et al., 2022; Yuan et al., 2024
Glycosylation	PTM via calnexin	Glycoprotein subunits	Stabilises receptor structure, trafficking	Potential target for enhancing receptor stability	Fukata and Fukata (2010)

* As a preprint, Hooda et al. (2024) present provisional findings pending peer review.

## 5 Pharmacological modulation and signalling ensembles

### 5.1 From uniform effectors to molecular ensembles

The classical view of GABA_A_Rs as uniform and monolithic mediators of inhibition has now given way to a more nuanced understanding: these receptors assemble into dynamic signalling ensembles. Within a single neuron, co-expressed receptor subtypes—each with unique ligand affinities, kinetics, and subcellular distributions—converge to produce finely tuned inhibitory regulation across space and time (Sente, 2024). This shift in perspective reframes GABA_A_Rs as adaptive molecular devices, capable of decoding the diverse chemical and electrical milieu of the brain.

**FIGURE 3:**
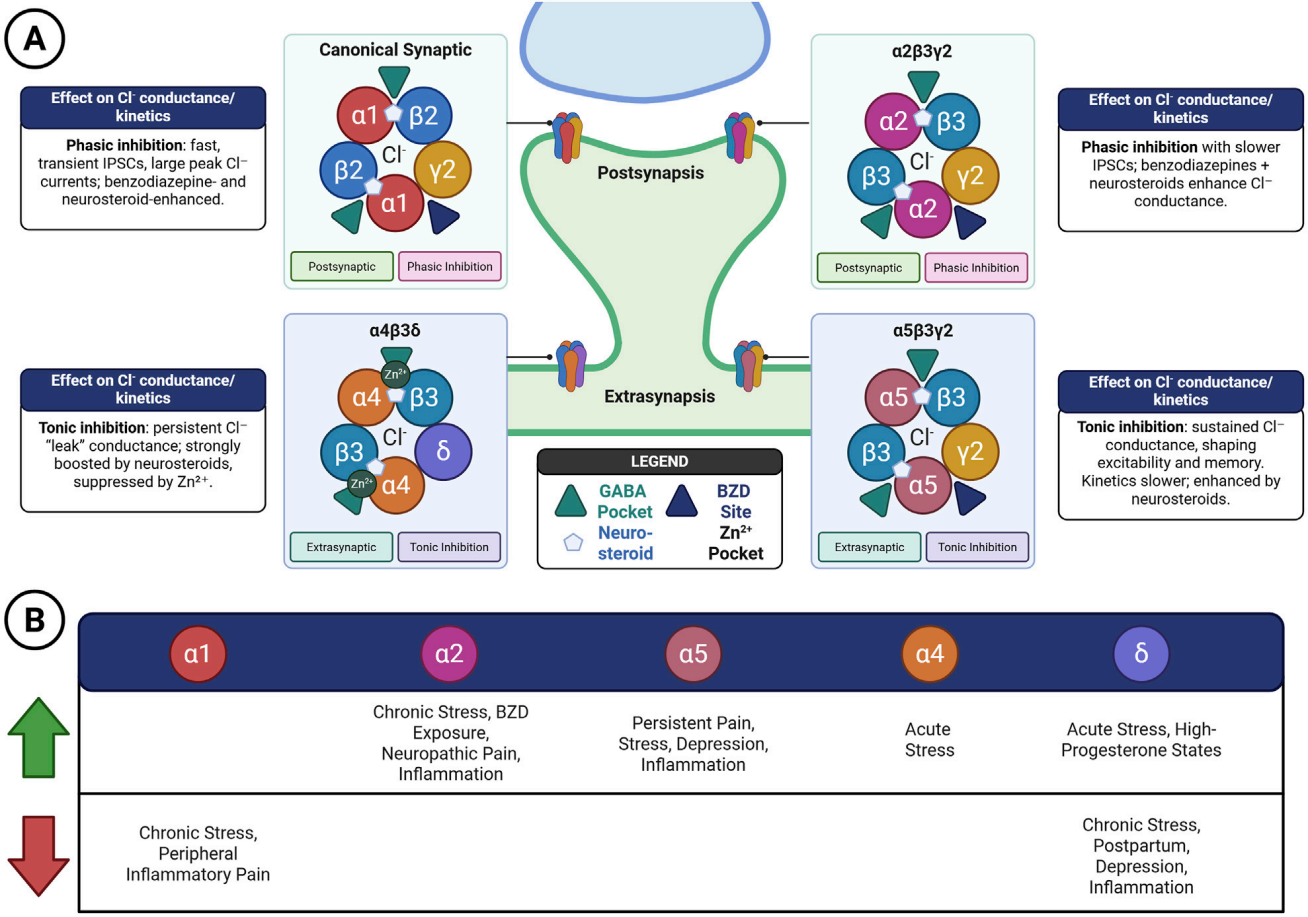
Ensemble logic of GABA_A_ receptor signalling. **(A)** The classical view of GABA_A_ receptors as uniform, monolithic mediators of inhibition has been replaced by the recognition that they form dynamic signalling ensembles. These ensembles consist of co-expressed receptor subtypes, each defined by distinct ligand affinities, kinetics, and subcellular localisation, thereby enabling nuanced regulation of inhibitory signalling across space and time (Sente, 2024). Within a single neuron, multiple GABA_A_ receptor configurations may coexist, allowing the cell to decode diverse extracellular cues—including GABA, histamine, neurosteroids, taurine, and netrin—as well as pharmacological agents such as benzodiazepines and endocannabinoids. Accessory complexes, such as TMEM132B–GABAA receptor, further illustrate the ensemble principle by modulating alcohol-related behaviours. **(B)** While α1-containing receptors remain prominent pharmacological targets, recent cryo-EM studies (Zhou et al., 2025; Sun et al., 2023) show that the β2–α1–β2–α1–γ2 stoichiometry predominates *in vivo*, underscoring the structural constraints of native populations and cautioning against assumptions of disease-specific selectivity. This heterogeneity generates distributed, input-specific inhibitory outputs, tuned to the physiological and computational demands of the circuit. Schematics were created with BioRender.com.

Importantly, multiple receptor configurations can coexist within a single cell, enabling neurons to interpret a range of extracellular cues. These include canonical neurotransmitters such as GABA itself, as well as histamine, neurosteroids, taurine, and netrin, along with pharmacological modulators like benzodiazepines (Rodríguez-Palma et al., 2023; Sente, 2024). The presence of accessory complexes, such as TMEM132B–GABA_A_Rs, further illustrates how receptor ensembles integrate endogenous and exogenous signals to influence behaviour, including responses related to alcohol consumption. Thus, rather than producing uniform inhibition, receptor heterogeneity creates distributed, input-specific inhibitory outputs that can be tuned to the computational and physiological demands of the circuit (Figure 3).

### 5.2 Adaptive modulation in physiological and pathological contexts

GABA_A_Rs ensembles are not fixed entities; they display remarkable plasticity, adapting to neuronal activity, hormonal fluctuations, inflammation, and developmental signals. In nociceptive circuits, for instance, α5- and α6-containing receptors play sex-specific and context-dependent roles. Upregulation of α5 subunits in the spinal cord exacerbates pain hypersensitivity, particularly in females, whereas α6 subunits may exert protective antinociceptive effects depending on cellular context (Franco-Enzástiga et al., 2021; Franco-Enzástiga et al., 2022; Rodríguez-Palma et al., 2023). In basal ganglia circuits, altered chloride homeostasis and the misassembly of receptor groups disrupt the pause–rebound firing of cholinergic interneurons, contributing to the motor pathology of Parkinson’s disease (Lozovaya et al., 2018). Similarly, aberrant subunit trafficking or misexpression underlies the hyperexcitability of epileptic networks and contributes to impaired synaptic development in neurodevelopmental disorders (Sieghart et al., 2022).

Despite these insights, critical mechanistic questions remain. How are subunits co-expressed and spatially targeted under diverse physiological conditions? What signalling pathways govern ensemble composition during development, stress, or disease? Can pharmacological agents be engineered to target ensemble components without destabilising overall inhibitory tone selectively? Most current therapeutics act at conserved binding sites, which limits their specificity and predisposes patients to side effects. Achieving precision in GABAergic modulation will require ligands that discriminate not only among subunits but also between conformational states, post-translational modifications, and microenvironmental contexts. Endogenous modulators, such as taurine (Jia et al., 2008) and netrin (Chan et al., 2022), along with accessory complexes like TMEM132B–GABA_A_Rs (Wang G. et al., 2024), underscore that a constant interplay between intrinsic and extrinsic signals tunes the ensemble composition. This complexity highlights both the difficulty and the necessity of achieving pharmacological specificity.

### 5.3 Endogenous regulation of ensemble composition

The composition of GABA_A_Rs ensembles is shaped by developmental cues, hormonal status, and environmental exposures, with distinct consequences across cell types and brain regions. For example, neonatal oligodendrocytes express α3, β2, and γ1 subunits, forming receptor ensembles that differ fundamentally from those in neurons and evolve dynamically over development (Ghit et al., 2021; Ordaz et al., 2021). Hormonal fluctuations, such as those occurring during pregnancy or across the oestrous cycle, alter the balance between γ2- and δ-containing receptors, thereby modulating anxiety levels and seizure susceptibility (Maguire and Mody, 2007; 2009).

Environmental exposures also reshape receptor ensembles. Ethanol provides a striking example: acute and chronic administration remodels surface expression of α4, δ, and γ2 subunits, altering both tonic and phasic inhibition and contributing to tolerance (Liang et al., 2007). In Alzheimer’s disease, region-specific shifts in GABA_A_Rs subunit expression are associated with impaired inhibitory control and progressive cognitive decline (Kwakowsky et al., 2018; Michałowski et al., 2025).

Endocannabinoids add yet another layer of endogenous regulation. The major central endocannabinoid, 2-arachidonoylglycerol (2-AG), directly potentiates β2-containing receptors at the transmembrane M4 site, independently of classical CB1/CB2 signalling. Its actions are synergistic with those of neurosteroids and benzodiazepines (Sigel et al., 2011). N-arachidonyl-glycine (NA-glycine), though less abundant, produces even stronger potentiation. It shares the β2 binding site with 2-AG but displays distinct kinetics and solubilisation properties (Baur et al., 2013). These observations highlight ensemble composition as a state-dependent and modifiable feature of neural circuits, one that is continually sculpted by endogenous molecules, hormonal environments, and external exposures.

## 6 Therapeutic targeting of extrasynaptic GABA_A_Rs

### 6.1 Extrasynaptic GABA_A_ receptors

Whereas synaptic GABA_A_Rs mediate fast, phasic inhibition, extrasynaptic GABA_A_Rs receptors sustain tonic inhibition by responding to low ambient concentrations of GABA. These receptors are characterised by high GABA affinity, minimal desensitisation, and distinctive subunit compositions that often involve α4, α5, α6, and δ subunits (Farrant and Nusser, 2005; Brickley and Mody, 2012; Lee and Maguire, 2014; Sieghart et al., 2022). Far from static entities, extrasynaptic receptors undergo continuous endocytosis and recycling between membrane compartments, redistributing dynamically between synaptic and extrasynaptic zones (Vien et al., 2016; Lorenz-Guertin and Jacob, 2018). These trafficking processes are fine-tuned by phosphorylation and other post-translational modifications (Connelly et al., 2013; Comenencia-Ortiz et al., 2014), allowing neurons to adjust tonic inhibition in response to physiological demands flexibly.

Among the best-characterised extrasynaptic subtypes are δ-containing receptors (for example, α4βδ and α6βδ). These receptors sustain tonic currents, regulate network excitability, resist blockade by classical antagonists such as bicuculline, and display sensitivity to neurosteroids and Zn^2+^ (Kasaragod et al., 2022). In parallel, α5-containing receptors, anchored at extrasynaptic sites through radixin–actin interactions, generate tonic inhibition in cortical and spinal neurons (Chong and Souayah, 2024; Orser, 2024). Disruption of radixin anchoring impairs receptor localisation and has been linked to perioperative cognitive disorders and neuroinflammation (Etherington et al., 2017; Lorenz-Guertin and Jacob, 2018; Dong et al., 2024; Orser, 2024; Wang Y.-J. et al., 2024; Zhang et al., 2025). Collectively, these receptors form a specialised non-synaptic inhibitory system with distinct structural, kinetic, and pharmacological properties that govern neuronal excitability and contribute to behaviours such as pain perception, sensory filtering, and stress adaptation.

### 6.2 Engineered cell models for studying extrasynaptic αβδ GABA_A_ receptors

Despite major advances in structural biology, extrasynaptic GABA_A_Rs—particularly those composed of αβδ subunits—remain technically challenging to study. Their low abundance, instability, and poor expression in heterologous systems have historically limited structural and pharmacological analysis (Hartiadi et al., 2016; Wongsamitkul et al., 2016).

To overcome these barriers, stable mammalian expression systems have been engineered to support reliable production and characterisation of α4β1/3δ receptors. One such system involves the stable expression of the human δ subunit in HEK293 Flp-In™ cells, co-transfected with α4 and β1 or β3 subunits at optimised ratios. This strategy yields functional α4β1/3δ receptors amenable to fluorescence-based membrane potential (FMP) assays in 96-well formats, enabling high-throughput pharmacological profiling (Falk-Petersen et al., 2017). δ-containing receptors in these systems can be distinguished by their selective responses to ligands such as THIP (gaboxadol), a δ-preferring superagonist, and DS2, a δ-selective positive allosteric modulator.

A complementary tetracycline-inducible expression system allows high-level production of human α4β3δ receptors, facilitating large-scale purification for structural studies (Zhou et al., 2018). These receptors exhibit pharmacological properties distinct from those of synaptic subtypes and play critical roles in mediating tonic inhibition, making them attractive therapeutic targets for epilepsy, anxiety, and sleep disorders. Together, these engineered platforms provide robust tools for dissecting extrasynaptic receptor biology and screening compounds that enhance tonic inhibition with reduced sedative liabilities.

### 6.3 Selective pharmacological agents targeting extrasynaptic GABA_A_Rs: Epilepsy

Extrasynaptic receptors are highly relevant to epilepsy, where tonic inhibition shapes neuronal excitability and seizure thresholds (Sierra-Paredes and Sierra-Marcuño, 2007). Because they mediate persistent currents in response to ambient GABA, these receptors stabilise network activity. However, during and after seizure episodes, they are particularly vulnerable to disruption through altered subunit expression, receptor internalisation, or changes in chloride homeostasis. These disturbances weaken tonic inhibition, lower seizure thresholds, and contribute to the development of recurrent hyperexcitability. In hippocampal CA1 neurons, for example, epileptiform activity induced by cyclothiazide is suppressed by the activation of extrasynaptic receptors, underscoring their role in seizure containment (Wan et al., 2014). Enhancing tonic inhibition may therefore provide a mechanism for seizure suppression that complements, rather than duplicates, phasic inhibition.

Epigenetic regulation also contributes to the function of extrasynaptic receptors in epilepsy. Silencing of the HDAC4 gene reduces seizure activity and improves cognition in rat epilepsy models, accompanied by increased expression of the α1 and α4 subunits, both of which are commonly associated with extrasynaptic locations (Zhang W. et al., 2019; Zhang et al., 2025). Similarly, inhibition of microRNA-155 reduces seizures by downregulating GABA transporters GAT-1 and GAT-3. This reduction in reuptake increases extracellular GABA, thereby enhancing activation of extrasynaptic receptors and strengthening tonic inhibition (Zhang Y. et al., 2019). These findings highlight epigenetic and transporter-based modulation as promising strategies for restoring inhibitory tone in epilepsy.

### 6.4 Selective pharmacological agents targeting extrasynaptic GABA_A_Rs: insomnia, stroke, angelman syndrome, and fragile X syndrome

Extrasynaptic receptors, particularly those composed of αβ subunits, are also implicated in conditions such as insomnia, stroke, Angelman syndrome, and Fragile X syndrome (Mortensen and Smart, 2006). Unlike synaptic receptors, these αβ assemblies exhibit low activation efficacy and heightened sensitivity to Zn^2+^ blockade. Structural and functional analyses show that Zn^2+^ inhibits αβ receptors by physically occluding the ion channel, even in the presence of GABA, thereby reducing chloride flux and dampening receptor activation (Kasaragod et al., 2022). These observations parallel earlier studies demonstrating Zn^2+^ sensitivity in δ-containing extrasynaptic receptors of the dentate gyrus, which are highly responsive to neurosteroids and implicated in epilepsy and memory disorders (Carver and Reddy, 2016; Chuang and Reddy, 2018; 2019). As outlined in Section 6.3, genetic and epigenetic interventions—including HDAC4 silencing and microRNA-155 inhibition—modulate extrasynaptic receptor function, highlighting their therapeutic relevance in epilepsy, stroke, and neurodevelopmental disorders. Together, these findings underscore the translational promise of selectively targeting αβ and αβδ extrasynaptic receptors to fine-tune tonic inhibition across diverse neurological conditions.

### 6.5 Extrasynaptic GABA_A_Rs in pain

Tonic inhibition mediated by extrasynaptic receptors also plays a crucial role in the processing of nociceptive signals. In the spinal dorsal horn, α5- and α6-containing receptors shape inhibitory tone in pain circuits, with their expression and function modulated by sex, inflammation, and injury (Delgado-Lezama et al., 2021; Franco-Enzástiga et al., 2021; 2022; Rodríguez-Palma et al., 2023). Chronic pain states in rodents are associated with upregulation of α5-containing receptors in the dorsal horn, correlating with increased excitability and mechanical allodynia. Pharmacological inhibition of α5-GABA_A_Rs reverses these pain behaviours, suggesting a pro-nociceptive role for α5 under certain conditions. In contrast, α6-containing receptors appear to suppress nociceptive transmission, promoting analgesia. This divergence highlights the significance of context-specific receptor composition in determining ensemble output. Sex-specific differences further complicate the picture. Female mice exhibit greater α5 expression in pain-relevant regions and heightened sensitivity to α5-selective modulators (Franco-Enzástiga et al., 2021). These findings align with broader evidence of sexually dimorphic organisation and plasticity in GABAergic circuits, which influences both susceptibility to chronic pain and responsiveness to therapies. Thus, tonic inhibition in nociceptive pathways emerges not as a static gain-control mechanism but as a dynamic process, tuned by receptor subtype, developmental stage, hormonal state, and inflammatory signals.

### 6.6 Extrasynaptic GABA_A_Rs in mood regulation

Beyond pain, extrasynaptic receptors influence mood regulation, particularly in the context of stress and depression. δ- and α5-containing receptors, enriched in hippocampal and cortical circuits, govern cognitive flexibility, emotional reactivity, and adaptive behaviours under fluctuating environmental demands (Bravo-Hernández et al., 2016; Feng et al., 2024; Guet-McCreight et al., 2024). Downregulation of δ subunits has been consistently observed in animal models of chronic stress and depression, leading to reduced tonic inhibition and increased limbic excitability. These deficits can be reversed by δ-preferring neurosteroids, such as allopregnanolone, which restore tonic inhibition and promote behavioural resilience (Maguire and Mody, 2007; 2009). α5-containing receptors similarly contribute to cognition and affect. In models of cognitive rigidity and learned helplessness, α5 activation restores hippocampal inhibition and behavioural adaptability, particularly when combined with antidepressant treatments (Botta et al., 2015; Prevot et al., 2019). Genetic or pharmacological manipulations of these subunits affect not only baseline mood states but also the efficacy of antidepressant responses. Collectively, these findings portray extrasynaptic GABA_A_Rs as homeostatic buffers, preventing excessive excitability in limbic and cortical circuits that regulate emotion and cognition. Their plasticity under stress highlights their therapeutic potential in mood disorders.

### 6.7 Selective pharmacological agents targeting extrasynaptic GABA_A_Rs: Depression

The therapeutic relevance of extrasynaptic receptors is particularly striking in depression, and especially in postpartum depression, where neurosteroid fluctuations disrupt GABAergic tone (Verbe et al., 2020; Feng et al., 2024). One agent, S44819, selectively inhibits α5-containing receptors and has demonstrated antidepressant efficacy in preclinical models (Etherington et al., 2017). In parallel, α5-preferring positive allosteric modulators (α5-PAMs) such as GL-II-73 exhibit antidepressant, anxiolytic, and pro-cognitive effects, while avoiding the sedative liabilities of diazepam (Botta et al., 2015; Prevot et al., 2019; Feng et al., 2024; Guet-McCreight et al., 2024). Although both GL-II-73 and S44819 display antidepressant-like efficacy, their mechanisms diverge. GL-II-73 primarily enhances synaptic α5 receptors, thereby supporting cortical resilience and plasticity, whereas S44819 preferentially inhibits extrasynaptic α5 receptors, thereby reducing maladaptive tonic inhibition associated with depressive states. Their context-dependent actions underscore the importance of receptor localisation, circuit state, and developmental stage in shaping therapeutic outcomes. Notably, GL-II-73 allows noninvasive monitoring of treatment efficacy via electroencephalographic (EEG) biomarkers. Computational models suggest that α5-PAMs normalise disrupted cortical processing by restoring spectral dynamics in EEG signals (Guet-McCreight et al., 2024), reinforcing their translational promise.

## 7 Toward a systems-level understanding of GABA_A_R ensembles

Taken together, current evidence compels a fundamental shift in how GABA_A_Rs are conceptualised: not as static inhibitory switches, but as modular, adaptive signalling ensembles. These ensembles weave together subunit diversity, assembly plasticity, membrane trafficking, and circuit-specific localisation to generate context-sensitive inhibition across the brain. Extrasynaptic receptors, particularly those incorporating δ, α5, and α6 subunits, exemplify this logic. Their activity is dynamically regulated by neuronal firing, hormonal status, and environmental stress, and their contributions to tonic inhibition are critical for setting the excitability thresholds of networks governing motor control, sensory integration, nociception, mood, and cognition (Farrant and Nusser, 2005; Belelli et al., 2009; Brickley and Mody, 2012; Comenencia-Ortiz et al., 2014). By anchoring inhibitory tone, these receptors function as adaptive buffers that shape the computational range of neural circuits.

From a therapeutic standpoint, the challenge is to move beyond broad-spectrum modulators and toward selective ligands that can reshape defined receptor ensembles without disrupting global inhibition. Such strategies would target receptors according to their subunit composition, stoichiometry, or membrane domain localisation, while also exploiting interventions that fine-tune assembly, trafficking, or degradation. To achieve this precision, molecular insights must be integrated with circuit-level data, linking receptor dynamics to network computation and ultimately to behaviour.

Emerging tools are poised to bridge this gap. Subunit-specific biosensors, cell-type–restricted pharmacogenetics, and *in vivo* imaging of inhibitory tone now make it possible to visualise receptor activity with unprecedented resolution. These approaches will clarify how inhibitory ensembles adapt across states and pathologies. Equally important is the recognition that GABA_A_Rs rarely operate in isolation: they participate in receptor–receptor interactions (Shrivastava et al., 2011), coupling with NMDA receptors, nicotinic acetylcholine receptors, and other neurotransmitter systems. This crosstalk expands the functional repertoire of inhibition, allowing inhibitory tone to be diversified across various contexts (Chai, 2025). Endogenous modulators, such as taurine (Jia et al., 2008) and netrin (Chan et al., 2022), along with accessory complexes like TMEM132B–GABA_A_Rs (Wang G. et al., 2024), further illustrate how ensembles integrate intrinsic and extrinsic cues into cohesive inhibitory outputs. These multilayered interactions underscore that inhibition is not a uniform process but a flexible computation, tailored to the demands of specific circuits and states.

Ultimately, a systems-level framework grounded in ensemble logic holds the potential to transform therapeutic strategies for neuropsychiatric disorders. Rather than relying on generalised suppression of excitability, the future lies in precision modulation—sculpting dynamic inhibitory architectures to restore balance where it has been lost, while preserving the adaptability that defines healthy brain function.

## 8 GABA_A_Rs in neural circuits

**FIGURE 4:**
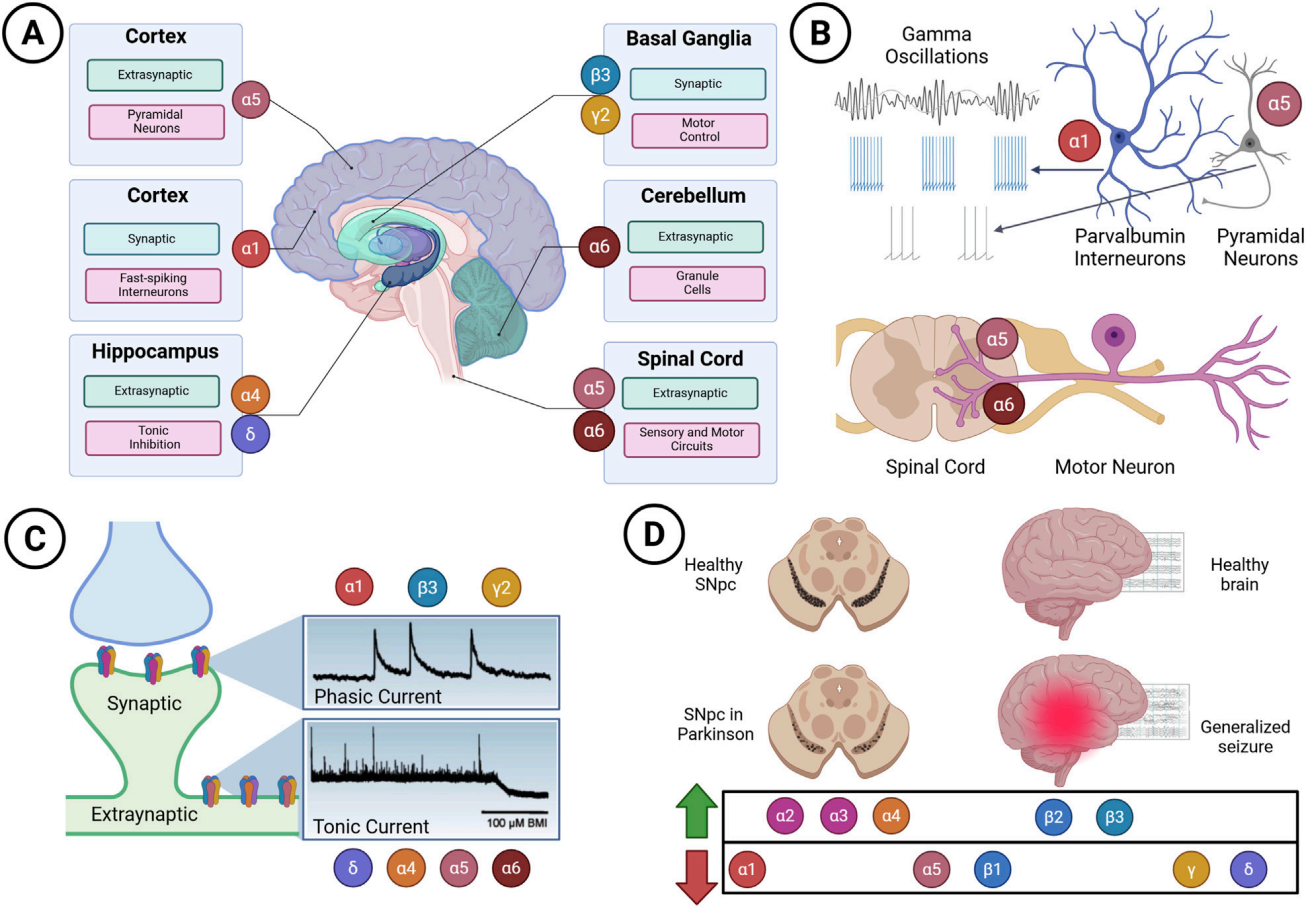
Circuit- and region-specific functions of GABA_A_ receptor subtypes. **(A)** Regional distribution of representative subunits across cortex, hippocampus, basal ganglia, cerebellum, and spinal cord. Canonical synaptic α1- and γ2-containing receptors dominate fast-spiking interneurons, whereas extrasynaptic α5-, α6-, and δ-containing receptors shape tonic inhibition in pyramidal neurons, granule cells, and spinal circuits. **(B)** Functional motifs linking receptor subtypes to circuit activity. α1-containing receptors in parvalbumin interneurons sustain gamma oscillations, while α5- and α6-containing receptors in motor and sensory pathways regulate spinal excitability. Crosstalk with NMDA and nicotinic acetylcholine receptors extends inhibitory modulation into broader network interactions. **(C)** Electrophysiological signatures of phasic (fast, transient inhibitory postsynaptic currents) and tonic (persistent “leak” currents) inhibition mediated by distinct receptor assemblies. Endogenous modulators, such as taurine and netrin, and accessory complexes, like TMEM132B–GABA_A_ receptors, further fine-tune these inhibitory modes. **(D)** Subunit-specific dysregulation in disease contexts, including altered expression in the substantia nigra pars compacta (SNpc) in Parkinson’s disease and cortical hyperexcitability in generalised seizures. Together, these examples underscore how subunit identity, localisation, receptor kinetics, and ensemble interactions shape inhibitory control across brain and spinal circuits, and how their disruption contributes to neurological disease. Schematics were created with BioRender.com.

The molecular diversity of GABA_A_Rs acquires its full physiological meaning only when placed within the context of neural circuits. Receptors are not uniformly expressed; instead, their subunit composition, subcellular localisation, and kinetic properties are selectively matched to the computational demands of defined neuronal types and circuit motifs. In practice, the brain deploys specific receptor assemblies—each with characteristic conductance, desensitisation kinetics, and pharmacology—at strategic cellular domains to achieve precise inhibitory control. The functional relevance of representative assemblies, their regional enrichment, and their association with neurological and psychiatric disorders are summarised in Table 4. In what follows, we examine how the diversity of GABA_A_Rs governs inhibitory signalling across cellular compartments, brain regions, and behavioural systems (Figure 4).

**TABLE 4:** GABA_A_ receptor assemblies and disease relevance.

Pentamer composition/Localisation	Brain region enrichment	Functional role	Associated disorders	Therapeutic strategies	References
α1β2γ2 (Synaptic)	Cortex, hippocampus, cerebellum	Fast phasic inhibition; sleep, sedation	Anxiety, epilepsy	Benzodiazepines, zolpidem	Masiulis et al., 2019; Olsen and Sieghart, 2008
α1β3γ2 (Synaptic)	Cortex, hippocampus	Phasic inhibition; gamma oscillations	Cognitive disorders, epilepsy	Benzodiazepines	Laverty et al. (2019)
α2β3γ2 (Synaptic)	Amygdala, spinal cord	Anxiety modulation, sensorimotor integration	Anxiety, chronic pain	α2-selective PAMs	Rudolph and Knoflach (2011)
α3β3γ2 (Synaptic)	Brainstem, hypothalamus	Developmental inhibition, circadian regulation	Sleep disorders	Barbiturates	Sigel et al. (1990)
α4β3δ (Extrasynaptic)	Thalamus, striatum, hippocampus (DG)	Tonic inhibition; seizure threshold regulation	Epilepsy, anxiety, and alcohol dependence	THIP, DS2, neurosteroids	Brickley and Mody, 2012; Wongsamitkul et al., 2016
α5β3γ2 (Perisynaptic/Extrasynaptic)	Hippocampus (CA1), cortex	Dendritic inhibition; cognition, memory	Cognitive impairment, depression	α5-NAMs (S44819), α5-PAMs (GL-II-73)	Prevot et al., 2019; Magnin et al., 2019
α5β3δ (Extrasynaptic)	Spinal cord, hippocampus	Tonic inhibition; pain modulation	Chronic pain, cognitive rigidity	α5 antagonists, HDAC4 silencing, miR-155 inhibition	Franco-Enzástiga et al., 2022; Zhang W. et al., 2019
α6β2/3δ (Extrasynaptic)	Cerebellar granule cells, DRG	Motor control, analgesia	Ataxia, tremor, migraine	α6-PAMs (compound 6-SL)	Sieghart et al., 2022; Rodríguez-Palma et al., 2023
α6β2γ2 (Synaptic)	Cerebellum	Inhibitory tone, precise timing	Motor disorders	Benzodiazepines	Boue-Grabot et al. (1998)
ρ1ρ2ρ3 (Synaptic GABA_C_)	Retina, cerebellum, spinal cord	Sustained inhibition; visual processing	Visual deficits, retinal diseases	ρ-subunit modulators	Barnard et al., 1998; Chuang and Reddy, 2018

### 8.1 Cellular precision in GABA_A_Rs expression

GABA_A_R subunit expression is tightly regulated at both cellular and subcellular scales, dictating receptor localisation (synaptic versus extrasynaptic) and biophysical profiles that, in turn, enable distinct inhibitory modes, such as phasic and tonic signalling, tailored to region- and cell–type–specific functions (Farrant and Nusser, 2005). Transcriptomic resources—RNA-Seq and microarray datasets from the Allen Institute—now enable the mapping of all 19 subunits across more than 100 brain regions (Ecker et al., 2025; Gillespie et al., 2025; Noh et al., 2025). Across the cortex, a relatively conserved profile emerges, dominated by α1, β2, and γ2, supporting a broadly uniform phasic inhibition. By contrast, limbic and brainstem structures—including hippocampus, amygdala, hypothalamus, cerebellum, pons, and medulla (myelencephalon)—exhibit marked heterogeneity: γ2, β3, and θ in hippocampus; β1 and γ1 in amygdala; ε and γ3 in hypothalamus; α6 and δ in cerebellum (Sequeira et al., 2019). This regional architecture underwrites specialised inhibitory strategies and signals concrete opportunities for region-selective pharmacology in neurological and psychiatric disease (Sequeira et al., 2019).

#### 8.1.1 Cortical and cerebellar circuits

In cortex and cerebellum, subunit composition is tuned by development, cell identity, and subcellular address, supporting both phasic and tonic inhibition. In the cortex, fast-spiking parvalbumin interneurons express α1-containing synaptic receptors that synchronise activity and support gamma rhythms, which are critical for cognition (Treviño, 2016). Development adds another layer: the early embryonic cortex preferentially expresses α3, α5, β3, and γ2. With maturation, α1, α2, α4, β2, and δ rise in region- and layer-specific patterns (Zavalin et al., 2024). The human and rodent cortex share common assemblies—α1/2/3 with β2/3 and γ2—and human embryonic stem cell–derived cortical neurons mirror embryonic rodent expression, with strong α2/3, β3, and γ2 (James et al., 2014).

In adulthood, laminar heterogeneity persists, with α1, α2, α5, and γ2 proteins distributing differentially across layers and cell classes (Sequeira et al., 2019; Zavalin et al., 2024). Demographic effects are evident in the human superior temporal gyrus: males show higher α1 than females; older females exhibit reduced α2, α5, and β3 compared to age-matched males; α3 is higher in young males versus older males (Pandya et al., 2019).

Cerebellar circuits display analogous precision. Extrasynaptic α5- and α6-containing receptors are abundant in pyramidal neurons and granule cells, where they mediate tonic inhibition to regulate excitability and input–output gain (Sieghart et al., 2022; Rodríguez-Palma et al., 2023). Granule cells, enriched for α6β2/3δ receptors, sustain persistent tonic currents that set baseline excitability (Bright et al., 2011). Purkinje cells express ρ1 along with α1, imparting GABA_A_R-like features to cerebellar inhibition (Boue-Grabot et al., 1998; Miller et al., 2017). Developmentally, the pig cerebellum illustrates a classic switch: prenatal α3 dominance gives way to postnatal α1, coinciding with the transition from depolarising to hyperpolarising GABA; chloride gradient maturation modulates α1, α3, and δ expression, reconfiguring polarity and strength of inhibition (Miller et al., 2017; Succol et al., 2012).

#### 8.1.2 Hippocampus and limbic regions

Hippocampal and limbic circuits deploy subunits in highly structured, dynamic patterns that shape inhibitory tone, plasticity, and disease vulnerability. In hippocampus, α1–3, α5, β2/3, and γ2 dominate (Serwanski et al., 2006; Glykys et al., 2008; Kerti-Szigeti and Nusser, 2016; Kwakowsky et al., 2018; Stefanits et al., 2018; Magnin et al., 2019; Palpagama et al., 2019; Ethiraj et al., 2021). Distribution is region- and layer-specific: α1 and α3 enrich dentate gyrus, CA1, and subiculum; α5 is relatively uniform but strong in CA1 pyramidal cells at both synaptic and extrasynaptic sites (Serwanski et al., 2006; Jacob, 2019; Magnin et al., 2019). β2/3 are present but faint in select layers (Stefanits et al., 2018); γ2 is widespread, conferring benzodiazepine sensitivity (Kwakowsky et al., 2018; Stefanits et al., 2018; Ethiraj et al., 2021). δ is concentrated in dentate granule cells, supporting robust extrasynaptic tonic inhibition (Glykys et al., 2008; Arslan, 2021).

Functionally, synaptic α1–3β2/3γ2 assemblies mediate phasic inhibition (Kerti-Szigeti and Nusser, 2016), whereas α5- or δ-enriched extrasynaptic receptors generate tonic inhibition (Serwanski et al., 2006; Glykys et al., 2008; Magnin et al., 2019; Arslan, 2021). Cell-type rules apply: α5 dominates CA1/CA3 pyramidal neurons, while δ is prevalent in dentate granule cells and molecular-layer interneurons (Glykys et al., 2008). Developmental dysregulation has a significant phenotypic impact—e.g., α4/δ disruption impairs tonic inhibition and is associated with epilepsy-like phenotypes, autism spectrum disorders, and deficits in temporal order memory and cognitive flexibility (Afroz et al., 2016; Smith et al., 2024). In Alzheimer’s disease, α1, α2, α5, and β2/3 are altered in region- and layer-specific patterns, destabilising inhibition and cognition (Killisch et al., 1991; Kwakowsky et al., 2018).

Limbic regions share this precision. In the amygdala, α1 is high in the lateral nucleus, α3 in the intercalated nuclei and subiculum, α5 and γ2 in the cortical nuclei and transition zones, while β2/β3 show low immunoreactivity (Stefanits et al., 2018). The entorhinal cortex expresses α1, α2, α3, α5, β2, β3, and γ2 with sex- and age-dependent variation (Ethiraj et al., 2021). Perturbations map to pathology: reduced expression or trafficking of β3- and γ2-receptors in amygdala and medial prefrontal cortex weakens inhibition and enhances fear memory—a core feature of PTSD (Huang et al., 2023). Thus, hippocampal–limbic heterogeneity underlies diverse roles in cognition, emotion, and disease.

#### 8.1.3 Spinal cord circuits

Spinal circuits express rich subunit repertoires across development (Sernagor et al., 1995; Chub and O'Donovan, 2001; Pflieger et al., 2002; Barrière et al., 2008; Yoon et al., 2010; Lu et al., 2014) and into adulthood (Delgado-Lezama et al., 2004; Grasshoff et al., 2008; Andres et al., 2014). Pharmacology with bicuculline, furosemide, and subunit-selective antagonists reveals functional extrasynaptic α4/α6- and α5-containing receptors in motoneurons and primary afferents (Bautista et al., 2010; Loeza-Alcocer et al., 2013). Furosemide’s selective block of α4/α6-mediated receptors enhances the monosynaptic reflex without altering dorsal root potentials—evidence for tonic inhibition at motoneurons—while α5 blockade depresses the dorsal root reflex (DRR) yet spares phasic afferent excitability, positioning α5 as a pain-modulatory target (Loeza-Alcocer et al., 2013).

Mechanistically, extrasynaptic α5 receptors depolarise motoneurons tonically in response to ambient GABA, reducing membrane resistance and setting spike thresholds (Loeza-Alcocer et al., 2013). At proprioceptive branch points, α5 receptors generate tonic primary afferent depolarisation (PAD), tune sodium channel availability, and stabilise spike conduction (Lucas-Osma et al., 2018). Segmental inhibition and sensory precision depend on α5 and α6 (Mahrous et al., 2025), and their blockade in non-human primates disrupts somatosensory processing, underscoring a vital role in ascending sensory gating (Mahrous et al., 2025).

#### 8.1.4 Basal ganglia and midbrain regions

In the basal ganglia and midbrain, differential expression of subunits supports motor and reward computations. In Parkinson’s disease, chloride dysregulation and receptor misassembly in striatal interneurons impair inhibition and motor initiation (Lozovaya et al., 2018). Neighbouring nuclei display distinct signatures: substantia nigra pars compacta (SNc) dopamine neurons favour α3 and γ2 with lower α1 and β2/3, whereas pars reticulata (SNr) GABAergic neurons show a different profile—evidence for fine-grained specialisation within close anatomical confines (Petri et al., 2002; Waldvogel et al., 2008; Waldvogel et al., 2009; Kwakowsky et al., 2018).

### 8.2 Circuit motifs and subunit-specific function

Canonical motifs recruit distinct receptor subtypes to sculpt timing and gain. Feedforward interneurons targeting pyramidal cells typically engage α1β2γ2 receptors, generating rapidly activating and deactivating phasic currents. Feedback inhibition and disinhibition often rely on α2/α3-containing receptors with slower kinetics, widening integration windows (Kasugai et al., 2010; Pelkey et al., 2017). Subtype identity correlates with short-term plasticity and drug sensitivity—α1 receptors desensitise faster and are zolpidem-sensitive; α2/α3 receptors desensitise more slowly and respond differently to benzodiazepines (Sieghart et al., 2022). These motif-level allocations evolve developmentally: an immature hippocampus relies on prolonged α2/α3-mediated inhibition to support early oscillations; maturation brings faster α1- and α5-linked synaptic control, sharpening temporal precision and modulating gain during associative learning (Botta et al., 2015).

### 8.3 Ensemble flexibility and circuit adaptation

GABA_A_Rs operate not as isolated entities but as ensembles—combinatorial mixtures whose composition shifts in response to cell state, activity, and network demands (Sente, 2024). This plasticity equips circuits to recalibrate inhibitory tone across development, environmental stressors, and injury. In the spinal cord, an ensemble configuration preserves fidelity: blocking GABAARs destabilises spike propagation at axonal branch points and diminishes sensory responsiveness at both spinal and cortical levels, implying a role for ensembles in safeguarding input transmission under high-frequency conditions (Mahrous et al., 2025). In the basal ganglia, loss of presynaptic inhibitory control drives exaggerated plasticity and motor sensitisation in Parkinsonian models, highlighting ensemble dysfunction—not merely receptor loss—as a mechanistic substrate (Borgkvist et al., 2015).

At the nanoscale, trafficking, diffusion, and clustering govern ensemble behaviour. Lateral diffusion, balanced by gephyrin anchoring, allows synapses to be remodelled by experience (Fritschy and Panzanelli, 2014; Cardin, 2019; Barberis, 2020). Activity-dependent modifications, such as phosphorylation and calcium signalling, tune receptor mobility and stability (Bannai et al., 2015). These dynamics let circuits toggle between inhibitory modes (e.g., hyperpolarising versus shunting), thereby supporting state transitions and adaptive plasticity (Cardin, 2019; Burman et al., 2023).

### 8.4 From receptors to behaviour: a translational bridge

A central translational aim is to link subunit composition to behaviour. Recent work shows that manipulating GABA_A_Rs subtypes reconfigures perception, learning, and motor output. In awake primates, spinal GABA_A_Rs blockade reduces thalamocortical responsiveness to somatosensory input, implicating these receptors in conscious sensory processing (Mahrous et al., 2025). In rodents, α5-containing receptors modulate memory and pain thresholds, while α6-preferring positive allosteric modulators enhance cerebellar and trigeminal inhibition without sedation (Bravo-Hernández et al., 2016; Sieghart et al., 2022; Rodríguez-Palma et al., 2023). Nevertheless, systemic pharmacology rarely achieves the spatial and subtype precision required for behavioural selectivity, underscoring the need to pair receptor-specific tools with circuit-targeted delivery and readouts that capture real-time inhibitory modulation.

### 8.5 Toward a circuit-aware inhibitory neuroscience

Realising the therapeutic potential of GABA_A_Rs diversity demands a shift from receptor-centric inventories to circuit-aware frameworks. GABA_A_Rs act within networks, where the timing, location, and extent of inhibition can be as consequential as the presence of specific subunits. Subunit identity, membrane targeting, and recruitment dynamics interact with circuit topology to shape inhibitory output. Accordingly, integrative methodologies are essential, including spatial transcriptomics and single-cell sequencing to chart expression, *in vivo* imaging and electrophysiology to track inhibitory tone across behaviour, development, and disease, and genetic, epigenetic, and optogenetic perturbations to causally dissect function within defined circuits and states.

Recent primate work illustrates this approach. Mahrous et al. (2025) demonstrated that spinal GABA_A_Rs blockade selectively disrupts evoked thalamocortical potentials while sparing baseline activity, supporting a circuit-specific role in sensory gating. The same receptors preserve reflex fidelity at axonal branch points, suggesting a presynaptic stabilising function (Mahrous et al., 2025). Such studies demonstrate that targeted manipulations not only reshape local synapses but also alter network computations and systems-level outputs.

The next phase is to model inhibition as a dynamic, context-dependent computation—regulated by neuromodulators, sculpted by epigenetic programs, and expressed via circuit-specific ensembles. Achieving this will require a synthesis of molecular profiling, real-time circuit imaging, and AI-driven modelling, enabling an inhibitory neuroscience in which each receptor’s role is interpreted within the architecture of behaviourally relevant networks.

## 9 Clinical implications and future directions

**FIGURE 5:**
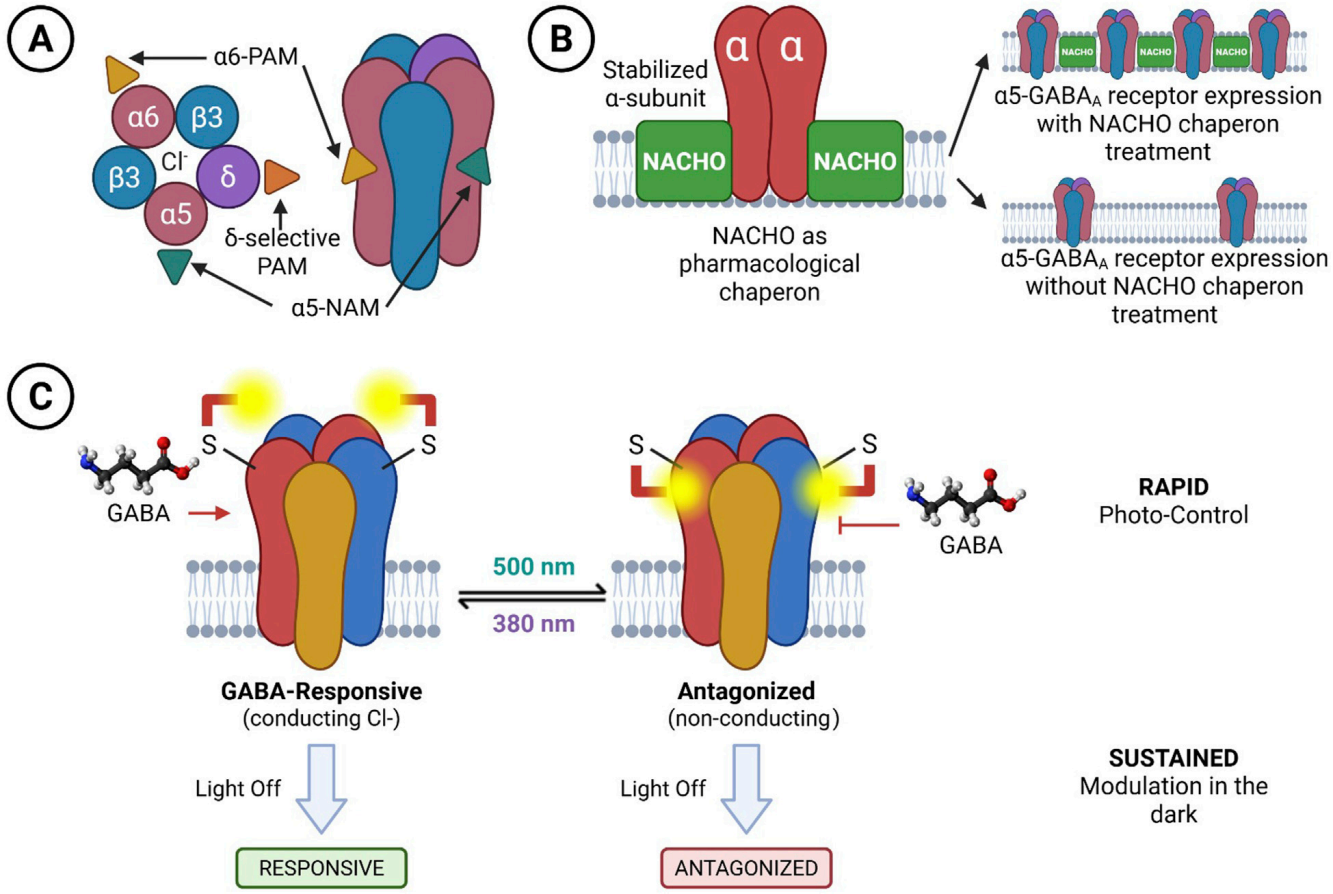
Emerging strategies for selective modulation of GABA_A_ receptors. **(A)** Pharmacological approaches targeting receptor subtypes. Selective positive allosteric modulators (PAMs) enhance α6- and δ-containing receptors, while α5-selective negative allosteric modulators (NAMs) reduce excitability in circuits implicated in pain, depression, and memory. **(B)** Pharmacological chaperoning. NACHO stabilises α-subunits during biogenesis, facilitating efficient surface expression of α5-containing receptors. Modulating this pathway offers a strategy to bias assembly toward therapeutically relevant subtypes (Hooda et al., 2024, preprint). **(C)** Optogenetic pharmacology. Photoswitchable ligands enable reversible, light-dependent control of receptor activity. Illumination at specific wavelengths rapidly toggles receptors between responsive (conducting) and antagonised (non-conducting) states, providing spatiotemporal precision and sustained modulation in darkness. Together, these strategies exemplify how pharmacological, endogenous (e.g., taurine, netrin), and synthetic biology tools—along with accessory complexes such as TMEM132B–GABA_A_ receptors—are advancing toward subtype-selective and circuit-specific control of inhibition. Schematics were created with BioRender.com.

The structural diversity and functional plasticity of GABA_A_Rs pose both a challenge and an opportunity for therapeutic development. Traditional GABAergic drugs, such as benzodiazepines, barbiturates, and certain anaesthetics, broadly enhance GABA_A_Rs activity across the central nervous system. While clinically effective, these agents lack subtype specificity, resulting in widespread inhibition that contributes to adverse effects, including sedation, tolerance, cognitive impairment, and addiction (Figure 5). This is the legacy model: strong symptom control at the cost of network-level precision.

The emerging model is different. In contrast, the ability to selectively target specific GABA_A_Rs subtypes offers a promising avenue for precision neuropharmacology. Among the most compelling candidates for therapeutic intervention are α5- and α6-containing receptors, which are defined by their anatomical localisation and specialised functional roles. α5- GABA_A_Rs, enriched in hippocampal and spinal circuits, regulate tonic inhibition and have been implicated in memory processing and pain hypersensitivity (Franco-Enzástiga et al., 2022; Rodríguez-Palma et al., 2023). α6- GABA_A_Rs, predominantly expressed in cerebellar granule cells and dorsal root ganglia, contribute to sensorimotor integration and provide anxiolytic and antinociceptive effects without inducing sedation (Sieghart et al., 2022). In other words, these receptors anchor circuit functions we care about—memory, pain, sensorimotor control—without the collateral sedation that undermines current care.

Subtype-selective allosteric modulators are advancing into translational frameworks. α5-selective negative allosteric modulators (NAMs) have shown efficacy in reducing anxiety and alleviating neuropathic pain. Conversely, α6-PAMs enhance cerebellar inhibition and diminish peripheral nociceptive transmission (Franco-Enzástiga et al., 2022; Rodríguez-Palma et al., 2023). These compounds preferentially act on circuits dominated by tonic inhibition, offering a therapeutic advantage in disorders where phasic transmission remains functionally intact but global inhibitory tone is dysregulated. This constitutes a paradigm shift—from “turning down the volume everywhere” to “equalising the band” at defined frequencies and nodes.

Additional strategies aim to manipulate GABA_A_Rs function at the level of subunit biogenesis and receptor assembly. One emerging idea is that modulation of the molecular chaperone NACHO could bias assembly toward therapeutically favourable subtypes (Hooda et al., 2024, preprint). Beyond chaperone modulation, gene-editing and RNA interference approaches offer the possibility of selectively downregulating maladaptive subunits (e.g., α5 in chronic pain) or upregulating beneficial ones (e.g., α6 in sensorimotor circuits) in a cell-type–specific manner. Target the factory, not just the product—an approach that could permanently re-weight ensemble composition.

Technological advances have accelerated the pace of structure-guided drug development. Artificial intelligence–driven cryo–electron microscopy pipelines now support atomic-resolution modelling of receptor–ligand interactions, ligand docking, and pharmacophore prediction (Sente, 2024). These tools enable iterative optimisation of compound design by identifying subtype-selective binding sites and predicting functional outcomes with increasing accuracy. Pharmacological precision is especially relevant for disorders such as Parkinson’s disease, where impairments in GABA_A_Rs subunit assembly and chloride homeostasis contribute to striatal disinhibition, motor dysfunction, and cognitive decline (Borgkvist et al., 2015; Lozovaya et al., 2018). Similar imbalances in receptor ensembles are likely involved in other circuit disorders, including epilepsy, schizophrenia, and chronic pain. Recent work demonstrates how the blockade of spinal GABA_A_Rs in non-human primates disrupts both local reflexes and thalamocortical sensory transmission, directly linking receptor-level modulation to altered perceptual and motor function (Mahrous et al., 2025). In addition to classical pharmacological agents, endogenous modulators such as taurine (Jia et al., 2008) and netrin (Chan et al., 2022), as well as accessory complexes like TMEM132B–GABA_A_Rs (Wang G. et al., 2024), further illustrate how ensemble composition can be tuned by intrinsic and extrinsic signals, thereby broadening the repertoire of potential therapeutic entry points. Precision will likely emerge from the intersection of structure, ensemble biology, and circuit context.

Innovative approaches in synthetic biology are also expanding the possibilities for circuit-specific inhibition. Optogenetic pharmacology tools, such as light-gated GABA_A_Rs (Li GABA_A_Rs) and photoswitchable GABA_A_Rs variants, enable precise, reversible control of native inhibitory synapses with millisecond resolution (Lin et al., 2015; Lopez et al., 2025). These tools enable real-time modulation of specific GABA_A_Rs populations during epileptiform activity or mood-related behaviours, opening new avenues for noninvasive therapeutic intervention. Think of them as “test drives” for circuit-targeted therapies—safe, reversible, and mechanistically clean.

Despite these advances, key translational barriers remain. Subunit expression is highly variable across species, developmental stages, sexes, and brain regions, complicating the extrapolation of preclinical results to human populations. Delivery systems for gene-based and chaperone-mediated interventions remain inefficient, particularly for deep-brain or spinal targets. Moreover, most subtype-selective compounds have yet to demonstrate consistent efficacy or safety in clinical trials. Overcoming these limitations will require integrating structural, functional, and spatial datasets to achieve a comprehensive understanding of the system. Single-cell transcriptomics and spatial proteomics will help chart receptor ensemble composition across regions and states. *In vivo* electrophysiology and imaging will validate the physiological roles of these ensembles. Crucially, modulation by environmental factors, such as inflammation, stress, and sex hormones, must be considered, as these can dynamically reshape subunit expression and drug sensitivity (Franco-Enzástiga et al., 2022; Rodríguez-Palma et al., 2023). Translation will succeed only if therapeutics are designed with state-dependence in mind.

Ultimately, the future of GABAergic therapeutics lies in the transition from global inhibition to targeted modulation. By embracing the complexity and ensemble logic of GABA_A_Rs signalling, it will become possible to recalibrate inhibitory tone with spatial, temporal, and functional precision. Essential tools, including subtype-selective modulators, structural modelling platforms, gene-editing systems, and intelligent delivery methods, are already in development. The next phase is to deploy them with mechanistic clarity, toward specific circuits, in well-defined pathological contexts. This vision must also incorporate receptor–receptor interactions (Shrivastava et al., 2011), such as functional coupling with NMDA and nicotinic acetylcholine receptors, as well as crosstalk with other neurotransmitter systems that diversify inhibitory outcomes (Chai, 2025). Together, these insights position ensemble-based GABAergic modulation not as an incremental improvement, but as a transformative strategy for precision therapeutics—moving the field from broad sedation toward circuit-informed restoration of function.

## 10 Conclusion

GABA_A_Rs are no longer to be viewed as static gatekeepers of inhibition. They are structurally diverse, dynamically regulated ensembles that decode inhibitory signals with remarkable spatial, temporal, and functional precision. This heterogeneity is not merely a consequence of 19 subunit genes; it emerges from the complex interplay of assembly rules, subunit stoichiometry, alternative splicing, post-translational modifications, and adaptive trafficking. These receptors are tailored to meet the computational needs of specific neuronal populations, governing excitability, gain control, synaptic plasticity, and ultimately determining how the brain balances signal integration with stability.

Recent advances in structural biology have catalysed this paradigm shift. High-resolution cryo–electron microscopy has uncovered previously unseen conformational states, novel ligand-binding pockets, and unexpected interactions with accessory proteins such as NACHO, which influence folding efficiency and subunit incorporation. At the same time, *in vivo* and *ex vivo* evidence shows that individual neurons co-express multiple GABA_A_Rs subtypes, forming functionally heterogeneous ensembles that integrate modulatory inputs from GABA, neurosteroids, histamine, and exogenous ligands. Work by Jonathan B. Cohen and colleagues has been particularly illuminating, defining the binding determinants of neurosteroids and showing how distinct sites mediate potentiation versus inhibition (Savechenkov et al., 2017; Wu et al., 2019; Yu et al., 2019; Jayakar et al., 2020). Endogenous modulators, such as taurine (Jia et al., 2008), guidance cues like netrin (Chan et al., 2022), and accessory complexes, including TMEM132B–GABAARs (Wang G. et al., 2024), exemplify how ensembles integrate intrinsic and extrinsic signals to diversify inhibitory control.

This ensemble logic reframes inhibition as context-sensitive and circuit-specific. Across the spinal cord, basal ganglia, cerebellum, and cortex, subunit composition dictates kinetic properties, neuromodulatory profiles, and pharmacological sensitivities. These configurations are dynamically regulated by development, activity, and behaviour. Crucially, pathophysiological states often arise not from GABA deficiency but from disruptions in subunit composition, receptor trafficking, or assembly. Such perturbations destabilise circuit dynamics and contribute to epilepsy, chronic pain, Parkinson’s disease, and schizophrenia. Moreover, interactions with other receptor systems—such as NMDA, nicotinic acetylcholine, and others—embed GABAAR ensembles into larger signalling frameworks that amplify their influence (Shrivastava et al., 2011; Chai, 2025).

For therapy, this heterogeneity is both a challenge and a profound opportunity. Classical GABAergic drugs deliver widespread inhibition, but with sedation, tolerance, and dependence as the inevitable tax. Emerging approaches promise greater precision: subtype-selective allosteric modulators, gene-editing interventions, assembly-biased chaperone manipulation, and AI-guided drug design. These strategies facilitate the selective recalibration of inhibitory tone at the levels of cell type, circuit motif, and behavioural state.

Still, the translational hurdles are formidable. Subunit expression varies by species, sex, developmental stage, and region, complicating the extrapolation from preclinical to clinical studies. Delivery systems for gene-based or molecular interventions remain inefficient for deep-brain and diffuse targets. Above all, we lack a comprehensive atlas of receptor ensemble dynamics across space, time, and behaviour. Without such a map, interventions risk treating inhibition as a blunt force rather than the finely tuned computation it is.

The next phase of inhibitory neuroscience must be unapologetically integrative. Molecular genetics, structural pharmacology, systems neuroscience, and behavioural physiology must converge. We need tools to track GABA_A_R subtypes *in vivo*, quantify ensemble configurations in real-time, and link these architectures to behaviour and disease. A circuit-aware paradigm—one that understands inhibition as a computation executed by dynamic receptor assemblies—will be indispensable.

Inhibition, then, is no longer a monolithic concept. It is a flexible, ensemble-driven computation encoded in receptor structure, expressed through circuit architecture, and modulated by internal and external states. Decoding and manipulating this logic with precision will define the future of inhibitory neuroscience. The next-generation of therapies will not be about dampening activity globally, but about rewriting the inhibitory code where it matters—at the level of ensembles that shape cognition, emotion, and behaviour.
